# The use of neuroimaging techniques in the early and differential diagnosis of dementia

**DOI:** 10.1038/s41380-023-02215-8

**Published:** 2023-08-22

**Authors:** Leonidas Chouliaras, John T. O’Brien

**Affiliations:** 1 Department of Psychiatry, University of Cambridge School of Clinical Medicine, Cambridge, UK; 2 Specialist Dementia and Frailty Service, Essex Partnership University NHS Foundation Trust, St Margaret’s Hospital, Epping, UK; 3 Cambridge University Hospitals NHS Foundation Trust, Cambridge, UK

**Keywords:** Neuroscience, Diagnostic markers

## Abstract

Dementia is a leading cause of disability and death worldwide. At present there is no disease modifying treatment for any of the most common types of dementia such as Alzheimer’s disease (AD), Vascular dementia, Lewy Body Dementia (LBD) and Frontotemporal dementia (FTD). Early and accurate diagnosis of dementia subtype is critical to improving clinical care and developing better treatments. Structural and molecular imaging has contributed to a better understanding of the pathophysiology of neurodegenerative dementias and is increasingly being adopted into clinical practice for early and accurate diagnosis. In this review we summarise the contribution imaging has made with particular focus on multimodal magnetic resonance imaging (MRI) and positron emission tomography imaging (PET). Structural MRI is widely used in clinical practice and can help exclude reversible causes of memory problems but has relatively low sensitivity for the early and differential diagnosis of dementia subtypes. ^18^F-fluorodeoxyglucose PET has high sensitivity and specificity for AD and FTD, while PET with ligands for amyloid and tau can improve the differential diagnosis of AD and non-AD dementias, including recognition at prodromal stages. Dopaminergic imaging can assist with the diagnosis of LBD. The lack of a validated tracer for α-synuclein or TAR DNA-binding protein 43 (TDP-43) imaging remain notable gaps, though work is ongoing. Emerging PET tracers such as ^11^C-UCB-J for synaptic imaging may be sensitive early markers but overall larger longitudinal multi-centre cross diagnostic imaging studies are needed.

## Introduction

Dementia, the umbrella term for global cognitive decline causing functional impairment, affects ~55 million people worldwide [1]. The most common types of dementia are Alzheimer’s disease (AD), Lewy Body dementia (LBD, a term which includes both dementia with Lewy Bodies (DLB) and Parkinson’s disease dementia), vascular dementia (VaD) and Frontotemporal dementia (FTD). There are several other less common forms of dementia such as Progressive supranuclear palsy (PSP), Corticobasal degeneration (CBD), Huntington’s disease, hippocampal sclerosis, prion disease and many others [2]. In all the degenerative dementias the onset of symptoms is associated with already established brain pathology, which develops many years before symptom onset [3, 4].

Early and accurate diagnosis of the cause of dementia is important for a number of reasons, including optimising clinical management, offering opportunities for secondary prevention, increasing prognostic accuracy and the identification of the right people to benefit from disease modifying therapies, once these become available [5–7]. Brain imaging by means of structural magnetic resonance imaging (MRI), at a single time point or performed serially, along with positron emission tomography (PET) imaging using ^18^F-fluorodeoxyglucose PET (FDG-PET), dopaminergic single-photon emission computerised tomography (SPECT) and PET imaging (for DLB) and PET ligands for amyloid and tau are the best established and validated imaging methods for both early and specific diagnosis of dementia subtype. Brain imaging along with cerebrospinal fluid (CSF) biomarkers have helped to establish the ATN (A = amyloid, T = tau, N = neurodegeneration) framework for the diagnosis of AD which is being used to define the presence of AD pathology at preclinical and prodromal stages [8]. This is of particular importance for the use of disease modifying treatments. This review summarises findings from diagnostic brain imaging studies, discusses novel developments in molecular imaging and outlines important future directions in the field.

## Structural MRI

Structural imaging with computerised tomography (CT) or MRI is widely used in clinical practice and recommended by several diagnostic and research guidelines for the assessment and diagnosis of people with dementia [8, 9]. MRI is preferred over CT when available. Structural imaging can exclude conditions such as space-occupying lesions, stroke, normal-pressure hydrocephalus, as well as many other pathologies and can also help with the differential diagnosis of dementia based on characteristic patterns of atrophy, white matter changes and the presence or absence of cerebrovascular disease [10]. The changes can be summarised in radiological reports and quantified using a variety of methods including visual assessment with validated rating scales, volumetric assessment using a region of interest approach or more detailed quantification using methods like voxel based morphometry or assessment of cortical thickness. It is however important to note that in these cases the role of neuroimaging is to corroborate a diagnosis based on identification of a clinical syndrome.

For example, in AD there is generalised atrophy with focal changes in the temporal lobe, especially the hippocampus. Early onset AD may be associated with more posterior and less temporal lobe atrophy. FTD is associated with anterior temporal pole and frontal atrophy, with semantic dementia subtype associated with asymmetric temporal atrophy. DLB shows relative preservation of the hippocampus and occipital and subcortical atrophy while vascular cognitive impairment and VaD are associated with cortical and subcortical vascular changes (latter including white matter hyperintensities (WMH), lacunes, enlarged perivascular spaces and microbleeds) [11, 12].

### Volumetric MRI

Visual assessment of scans using rating scales are reliable and offer diagnostic accuracy equivalent or better to unstructured scan evaluation by expert raters with an area under the curve (AUC) ranging from 0.67 to 0.97 for the differential diagnosis of different types of dementia [11, 13]. Considering automated volumetric analyses, structural MRI atrophy maps to identify patterns characteristic for AD, LBD or FTD showed that these atrophy maps had 90% sensitivity and 84% specificity for AD, 78.7% sensitivity and 98.8% specificity for LBD and 84.4% sensitivity and 93.8% specificity for FTD [14]. A study that included 504 individuals with AD, FTD, VaD, DLB and control subjects, quantifying volumetric, morphometric and vascular characteristics showed that MRI was accurate in 70.6% of cases. VaD groups were detected with 96% sensitivity, controls with 82%, and AD with 74%. DLB was the most difficult for detection with 24% sensitivity [15]. Koppel et al. compared 134 cases with AD, FTD, LBD and MCI and were able to separate healthy elderly from patients with dementia with an AUC of 0.97 [16]. Ma et al. found that a proposed deep learning framework achieved an overall accuracy of 88.28% in differentiating AD from FTD [17] while Yu et al. showed that an AD atrophy index could identify AD and FTD from controls with an AUC of 0.88 and an FTD atrophy index could identify FTD from AD with an AUC of 0.93 [18].

**Fig. 1:**
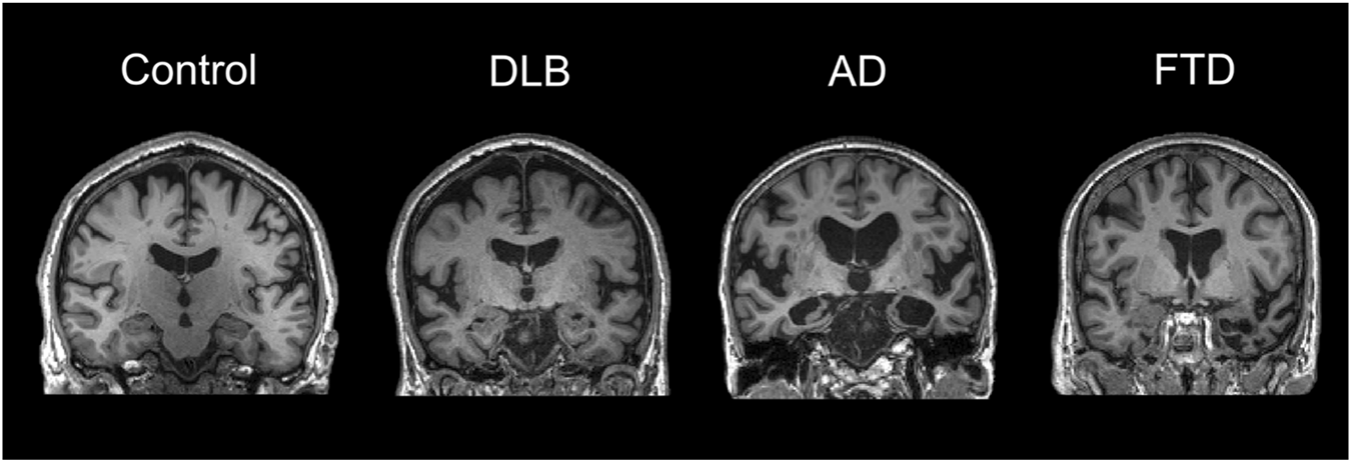
Representative MRI scans in different types of dementia. The figure shows representative MRI scans from a non-demented control and from patients with Dementia with Lewy Bodies (DLB), Alzheimer’s disease (AD) and frontotemporal lobe degeneration (FTLD). It highlights the characteristic patterns of atrophy with relative preservation of the hippocampus in DLB, severe hippocampal atrophy in AD and temporal pole atrophy in FTLD. These scans are from the Neuroimaging of Inflammation in Memory and Other disorders (NIMROD) study cohort. Images are courtesy of Dr Elijah Mak, University of Cambridge, UK.

In terms of structural changes in LBD, brain atrophy is seen but the relative preservation (compared to the marked atrophy in AD) of cortical structures and the medial temporal lobe is well established (Fig. 1) and is a supportive feature in the diagnostic criteria for DLB [19–21]. Mak et al. compared 35 DLB, 36 AD and 35 controls and suggested that the relative preservation of the hippocampus in DLB is characterised by preservation of the cornu ammonis (CA) 1, fimbria and fissure while all other hippocampal subfields had comparable atrophy in both AD, DLB and control groups [22].

Nevertheless, assessment of atrophy in older populations can be more challenging. Barkhof et al. carried out post-mortem MRI in 132 autopsy brain tissues from the Vantaa 85+ community study and compared visual ratings of medial temporal lobe atrophy (MTA) to neuropathological findings [23]. Overall, high MTA scores were associated with clinical dementia with sensitivity of 63% and specificity of 69% for AD [23].

In terms of use of MRI in earlier dementia stages, a review summarising 33 studies in structural neuroimaging for the early diagnosis of AD in people with MCI (including 3935 participants) concluded that there is lack of systematic approach in data collection, analysis and interpretation [24]. Using machine learning to quantify neurodegeneration patterns in structural MRI, studies have managed to predict MCI conversion to AD with modest accuracy ranging from 63 to 85% depending on the cohort, imaging modality and models used [25–27].

In a study focusing on prodromal DLB, Kantarci et al. compared 56 patients with MCI and features of DLB to 112 cognitively unimpaired controls. They showed that at baseline prodromal DLB was associated with atrophy in the nucleus basalis of Meynert, measured through region of interest analysis from an in-house atlas of the substantia inominata [28].

Overall, a large body of evidence suggests that volumetric analyses on MRI have an important role for the differential diagnosis of dementia with high specificity when changes are present, especially using automated analyses. Volumetric MRI changes however lack sensitivity in early prodromal dementia stages as they mostly correlate with established neurodegenerative processes. It is important to highlight that research studies generally utilise well characterised participants recruited at clinical academic settings enhanced for patients with a more clear-cut diagnosis following certain inclusion and exclusion criteria set out by each study and therefore the quoted calculated sensitivities and specificities are likely to be overestimated with regards to real world patient settings. There is a high likelihood of co-pathology in patients with dementia and often patients with mixed dementias may show neuroradiological features characteristic of more than one type of dementia, e.g. atrophy and infarcts.

### White matter hyperintensities and cerebral microbleeds

A substantial burden of WMH, lacunes and strategic infarcts are consistent with vascular cognitive impairment and dementia [29]. Nevertheless, there is a significant association between WMH, grey matter atrophy and cognitive decline in AD and FTD. Dadar et al. compared 571 normal aging subjects with 551 MCI, 212 AD, 125 FTD and 271 PD from the Alzheimer’s disease neuroimaging initiative (ADNI), the frontotemporal lobe degeneration neuroimaging initiative and the Parkinson’s Progression Markers Initiative datasets [30]. They found significantly higher WMH loads in MCI, AD and FTD compared to controls. WMH were related to grey matter atrophy in insular and parieto-occipital regions in MCI/AD and frontal regions and basal ganglia in FTD. WMH were associated with more severe cognitive deficits in AD and FTD but had no impact in MCI and PD. Importantly, WMH are associated with higher cardiovascular risk factors in midlife [31, 32]. However, they have also been linked to tau pathology, a reminder that WMH cannot always be taken to represent vascular disease [33].

Cerebral microbleeds are common in people with AD, DLB, stroke and trauma [34]. They represent iron accumulation in perivascular spaces and are linked with vascular disease and cerebral amyloid angiopathy [35]. Lobar microbleeds are associated with amyloid pathology while deep/basal ganglia microbleeds are associated with hypertensive small vessel disease [36, 37]. Their role in the pathophysiology and diagnosis of different types of dementia is not yet clear but they were found to be of similar frequency among patients with AD and DLB, albeit with greater densities in the parietal, temporal and infratentorial regions in AD compared to DLB [38]. Meanwhile in patients with first episode ischaemic stroke, three or more microbleeds were associated with higher risk of developing vascular dementia [39]. Studies in younger and presymptomatic individuals showed that cerebral microbleeds are significantly higher in number in APOE ε4 carriers [40]. Studies in unimpaired populations that were longitudinally followed up showed that high microbleed number (>3–4) is associated with an increased risk of cognitive deterioration and dementia [41, 42].

### Serial MRI

Serial MRI has been used as a measure to improve differential diagnosis of dementias and has often been incorporated as a secondary outcome measure in clinical trials in AD. It is well established that serial atrophy rates are significantly higher, approximately fourfold, in AD compared with similarly aged controls. Rates are also higher than controls in VaD and FTD. One study found people with AD had an atrophy rate of 2.0% per year compared to 1.9% in VaD and 1.4% in DLB [43]. Further studies have also shown greater atrophy rates in AD compared to DLB which showed similar atrophy rates to controls, a finding in keeping with the lesser overall atrophy in DLB [44]. In parallel, studies have shown that DLB with co-existing AD pathology is associated with faster rates of progression suggesting that the presence of AD pathology is likely the driver of atrophy [45, 46]. In a study comparing behavioural variant (bv)FTD, AD and healthy controls with consecutive scans over at least 12 months, Frings et al. showed that annual volume decline was larger in bvFTD, then AD, and then in controls, predominantly in white matter of temporal areas and orbitofrontal grey matter [47]. In summary, studies in longitudinal atrophy in dementia can support a specific diagnosis but considering the interval required between scans and the lack of sensitivity may not be as useful for early diagnosis.

## Diffusion weighted imaging MRI

Diffusion tensor imaging (DTI) is an MRI technique that provides information on the orientation and integrity of white matter tracts through measuring parameters associated with diffusion of water molecules in the brain. It generates measures of fractional anisotropy (FA) and mean diffusivity (MD) of water molecules in a region of interest, and studies have shown lower FA and higher MD (associated with reduced axonal integrity) in MCI and AD compared to controls [48, 49]. DTI data are also used for other analytical methods such as tractography to investigate tract integrity. Compared to AD, FTD was associated with lower FA in frontal regions [50] while specific tractography of long and short white matter tracts suggested that large scale tracts are particularly vulnerable to vascular disease in FTD and associated with executive dysfunction while short tracts were associated with semantic symptoms [51]. DTI differences may be able differentiate typical AD from the posterior cortical atrophy variant of AD showing differences in regions including parietal and temporal lobe areas [52]. Meanwhile, DLB was associated with increased amygdala MD compared to AD [53]. Spotorno et al. compared 34 LBD patients with 16 PSP and 44 healthy controls using a FA score from a combination of regions sensitive to pathologic features of PSP [54]. They distinguished PSP from LBD with AUC of 0.97 with sensitivity of 0.94 and specificity of 0.91. They validated these results in a second cohort with 34 patients with PSP, 25 LBD and 32 controls with an AUC of 0.96 [54].

In a study using DTI data for tractography analyses in the nucleus basalis of Meynert (NBM), Schumacher et al. compared the cholinergic white matter pathways in 46 AD, 48 DLB 35 MCI-AD, 38 MCI-LB and 71 control participants and found that MD of the lateral pathway was higher in the dementia and MCI groups and that particularly in MCI, loss of integrity of both NBM pathways was associated with an increased risk of progression to dementia [55]. Recent novel studies assessing cortical microstructure via cortical mean diffusivity (cMD) were found to be more sensitive than macrostructural neurodegeneration. Along with free water fraction (FW), cMD changes have shown that in the AD continuum, microstructural changes show a biphasic trajectory. There is increased cortical thickness and decreased cMD and FW in the initial presymptomatic dementia stages while there is decreased cortical thickness and increased cortical MD and FW in symptomatic changes [56]. cMD was found to be associated with PET tau in vulnerable to AD pathology regions and predict hippocampal atrophy rate and cognitive decline while cortical microstructure changes in the frontal and parietal areas appeared to be sensitive biomarkers for microstructural alterations in FTLD subtypes [57–59].

In summary, DTI has been successfully used in research studies to show biologically plausible differences between dementia subtypes and to predict progression from MCI to dementia. However, studies have been modest in size and from single sites, and no clearly established cutoffs or harmonised, validated methods are available, limiting the ability for DTI to be used in clinical practice.

## Assessment of blood flow and perfusion

MRI can be used to measure blood flow, either through the use of injected contrast agents or through magnetically labelling blood, a technique known as arterial spin labelling (ASL). Blood flow closely matches the patters of hypometabolism on FDG-PET due to the close coupling between perfusion and metabolism in brain [60, 61]. ASL was shown to be comparable to FDG-PET in identifying AD compared to controls with an AUC of 0.91 [62]. However, in a study using PET-MR that compared FDG-PET with ASL, Ceccarini et al. compared a combined group of 27 patients with AD, DLB, FTD and 30 matched controls and found that FDG-PET performed better than ASL [63]. In keeping with patterns on FDG-PET, DLB has been associated with reduction in cortical perfusion on ASL in higher visual areas compared to AD [64]. Such findings were similar in a cohort with MCI-LB with reduction in posterior parietal and occipital regions but relatively preserved posterior cingulate [65].

Using ASL in 32 early onset AD and FTD patients and 32 controls, ASL achieved an AUC of 86–91% for the correct classification of patients with dementia and potentially adds diagnostic value when combined to structural MRI data [66]. In an attempt to differentiate early AD from bvFTD, Stekeete et al. compared 13 AD with 19 bvFTD and found that AD was associated with hypoperfusion in the posterior cingulate cortex and this differentiated, to some extent, AD from bvFTD though AUC was modest at 0.74 [67]. In a comparison of ASL with FDG-PET in ten FTD patients and ten controls, Anazodo et al. found that FDG-PET outperformed ASL in inter-rater reliability as well as sensitivity and specificity in discriminating patients from controls (ASL AUC 0.75 and FDG-PET AUC 0.87) [68]. ASL findings however in AD and FTD are not consistent, with an earlier study by Du et al. in 21 FTD, 24 AD and 15 controls showing that FTD and AD display different spatial patterns of hypoperfusion on ASL and were able to classify AD from FTD with an AUC of 0.87 [69].

In combining DTI with ASL to differentiate early onset AD with early onset FTD, Bron et al. compared 24 AD and 33 FTD with 34 controls and used support vector classification finding that ASL and DTI combined with structural MRI could differentiate AD from FTD with AUC 0.84 compared to structural MRI alone with AUC of 0.72 [70]. ASL has further shown some promise in the differential diagnosis between AD and DLB with distinct patterns seen in DLB compared to AD and cognitively normal individuals [71, 72].

In studies focusing on the blood brain barrier (BBB), dynamic contrast-enhanced MRI with temporal and spatial resolutions to measure BBB permeability have shown a breakdown of the BBB in the hippocampus of patients with early cognitive dysfunction, independent of their amyloid and tau biomarker status and this also occurs in normal aging [73, 74]. In a follow up study, BBB breakdown in the hippocampus and medial temporal lobe was able to distinguish Apolipoprotein (APOE) ε4 from non-ε4 carriers [75].

In summary, studies in blood flow and perfusion in dementia have shown great potential for the early detection of neurodegeneration. However, the differences detected are subtle and multi-centre studies in large cohorts are lacking in order to test their potential use in clinical practice. FDG-PET seems to outperform methods for MRI cerebral blood flow and is more widely adopted.

## Functional MRI

Functional MRI measures blood flow changes to determine which parts of the brain are engaged when performing a test or at rest and can study brain networks that are functionally connected [76]. The default mode network (DMN) activity has been found to be abnormal in AD [77–79]. Resting state fMRI has been used for the differential diagnosis between AD and bvFTD with decreased connectivity in the lateral visual cortical network, lateral occipital and cuneal cortex as well as the auditory system network and angular gyrus seen in bvFTD compared to decreased connectivity in the dorsal visual stream network and lateral occipital and parietal cortex in AD [80]. The disrupted functional connectivity seen in AD has been associated with tau burden and neuroinflammation measured through in vivo PET imaging [81, 82].

In DLB, functional connectivity has been used to study symptoms of cognitive fluctuations. Peraza et al. found that the DMN is unaffected in DLB compared to controls but DLB patients show differences in the left fronto-parietal, temporal and sensory motor-network suggesting a potential al role of attention-executive networks in the aetiology of cognitive fluctuations in DLB [83]. Recently, it has been suggested that higher physical activity is associated with greater connectivity in the DMN and may be one of the pathways through which exercise promotes resilience to neurodegeneration [84]. Overall studies in functional MRI in dementia have shown differences in resting state functional connectivity and have pointed to specific networks and regions affected in each dementia, however there seems to be a significant overlap among diagnostic groups and unlikely to be useful clinically.

## Magnetic resonance spectroscopy, electroencephalography and magnetoencephalography

Magnetic resonance spectroscopy, an MRI method measuring metabolite levels in the brain, has been explored in dementia research. The present findings suggest the need for larger studies with more consistent methodology before being considered for use in clinical practice (for systematic review of studies please see [85] Similarly, studies using electroencephalography and magnetoencephalography for the differential diagnosis of dementia are lacking and more research is needed for these important imaging modalities [86, 87].

## More advanced MRI methods for the diagnosis of dementia

More advanced ways of brain MRI imaging, for example the neurite orientation dispersion and density imaging (NODDI) have shown great promise for the early and differential diagnosis of dementia. NODDI is a DTI technique that derives measures of orientation dispersion index and neurite density index and can detect distinct microstructural features [88]. NODDI changes have been shown as part of brain aging and seem to complement traditional DTI measures by characterising the cytoarchitecture of brain tissue [89–91]. In dementia, NODDI has been studied in young onset AD [92, 93] showing it is affected in regions associated with early atrophy in AD while NODDI measures in animals correlate with tau burden [94]. NODDI measures seem to be lower in temporal and parietal cortical regions in MCI when compared to controls while they are lower in parietal, temporal and frontal regions in AD [95]. In a multi-centre study, Raghavan et al. tested the associations between NODDI and neuropathological changes in the Mayo Clinic Study of Aging and the Mayo Alzheimer Disease research centre cohorts and found that cerebrovascular disease, tau and TDP-43 pathologies cause white matter microstructural damage seen with the NODDI methods [96]. NODDI however as an emerging new imaging method maybe subject to biases, e.g. presence of CSF partial volume in individuals with larger ventricles or atrophy due to degeneration [97]. While NODDI is a very promising new method of DTI, there are no studies yet looking specifically at its potential at the early presymptomatic diagnosis of dementia or its potential role in the differential diagnosis of the most common types of dementia.

Most MRI studies today have been done in the widely available 1.5 and 3 Tesla scanners. New technologies allow for higher power magnet and 7 Tesla MRI (7T) scanners are now becoming more widely used and allow higher signal-to noise resolution. 7T studies have measured hippocampal subfield volumes in AD and imaged the substantia nigra in PD [98]. Van Rooden et al. showed that increased cortical phase on 7T may reflect early stages of amyloid beta (Aβ) pathology in AD [99] while Theyshon et al. found that the use of 7T in vascular dementia may be more sensitive in the detection of cerebral microbleeds [100]. 7T MRI imaging has a huge promise in research and clinical practice but there are still challenges with regards to the costs, operating complexity, and availability, while diagnostic superiority for dementia over lower field strength MRI remains to be shown [101].

## ^18^F-Fluorodeoxyglucose (FDG) PET

FDG-PET changes are a supportive feature in the AD and DLB diagnostic criteria and FDG-PET is widely used clinically for the diagnosis of AD and the differential diagnosis of different subtypes of dementia [21, 102, 103]. FDG-PET is a readout of the local cerebral metabolic rate of glucose consumption [104]. Reduced uptake of the radioactive compound is suggestive of hypometabolism which in the brain correlates with reduced synaptic activity and evidence of neurodegeneration, correlating with brain atrophy and tau pathology [101, 105]. It is analysed either using expert visual rating or specialised quantitative analytical software [106–108]. Meta-analytic evidence suggests that FDG-PET has 90% sensitivity and 89% specificity in diagnosing AD from controls [109]. FDG-PET was found to have superior diagnostic accuracy in AD and DLB compared to hexamethylpropyleneamine oxime (HMPAO) SPECT with an AUC of 0.93 for FDG-PET compared to 0.72 for HMPAO SPECT [110]. A recent systematic review by Fink et al. analysed the accuracy of FDG-PET comparing AD to non-AD dementias and showed a median sensitivity of 0.89 and specificity of 0.74 [111].

**Fig. 2:**
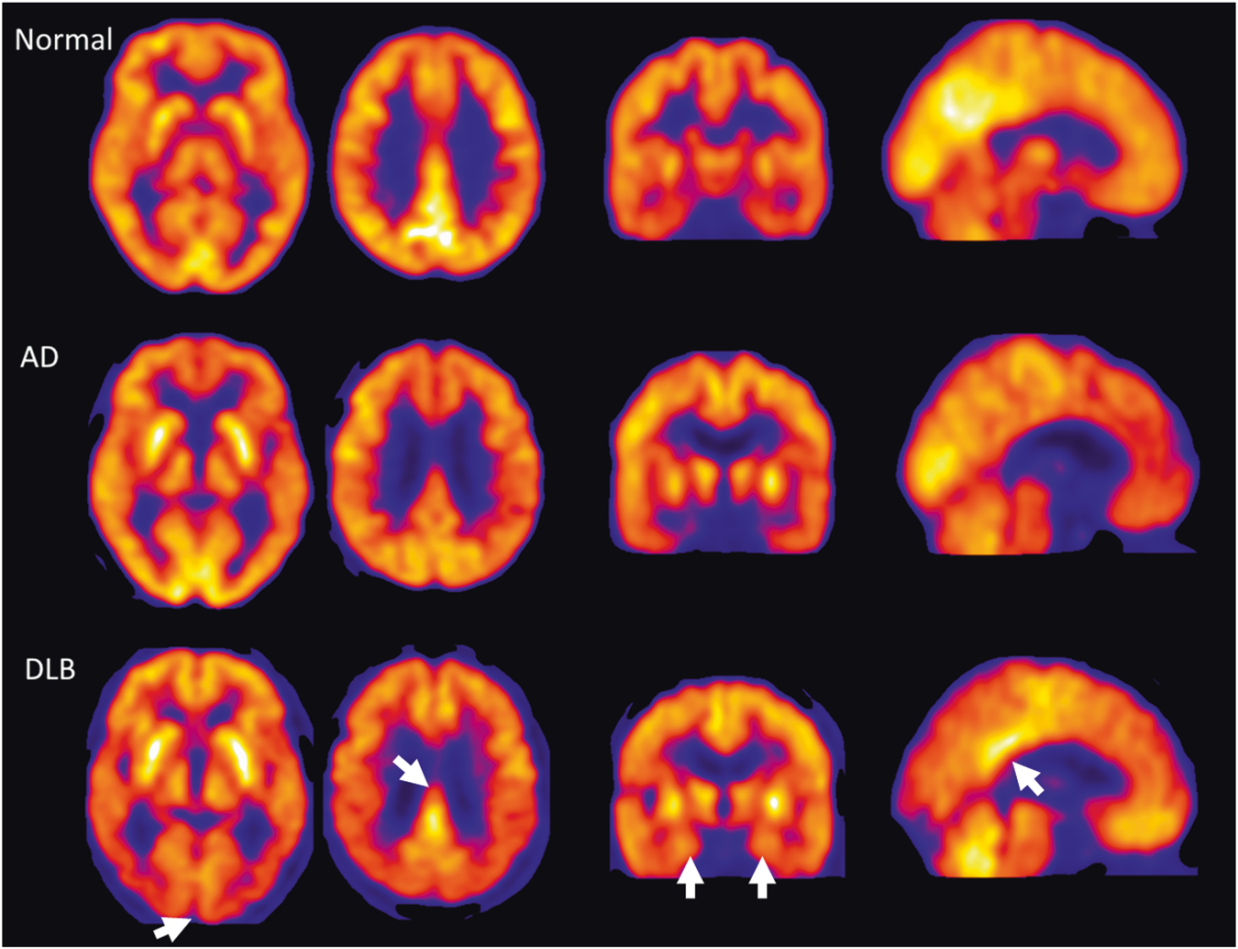
^18^F-Fluorodeoxyglucose (FDG) PET in Alzheimer’s disease (AD) and Dementia with Lewy Bodies (DLB). FDG PET representative images showing reduced local cerebral metabolic rate of glucose consumption in cases of AD and DLB compared to a non-demented control study participant. The white arrows highlight the relative preservation of the hippocampus and posterior cingulate gyrus in DLB compared to AD and the occipital hypometabolism in DLB. These are FDG PET scans from the Study of the clinical utility, patient preference and cost benefit of SPECT and PET-CT brain imaging in the evaluation and diagnosis of Alzheimer’s Disease (Suspected-AD). Images are courtesy of Dr Michael Firbank, Newcastle University, UK.

The patterns seen in AD involve hypometabolism of the temporal and parietal lobes (Fig. 2). In dominantly inherited AD, hypometabolism on FDG-PET can be detected as early as 10 years before symptom onset [112]. There is evidence that FDG-PET may also predict conversion from MCI to dementia however limitations relating to individual studies with small sample sizes do not allow reliable meta-analyses of such studies and pooled results show large range in the sensitivity (56–100%) and specificity (24–100%) for the role of FDG-PET in predicting conversion from MCI to dementia [113, 114]. Considering the variability of FDG-PET it is therefore not recommended for clinical use at the MCI stage [115].

Using FDG-PET data from the ADNI, Blazhenets et al. showed that FDG-PET in combination with amyloid PET and non-imaging variables may improve the prediction of conversion from MCI to AD and support the stratification of patients according to their conversion risks [116]. Levin et al. used FDG-PET in the ADNI dataset to subtype AD in ‘typical’, ‘limbic-predominant’ and ‘cortical-predominant’ types that correlate with the brain atrophy subtypes and the different clinical trajectories [117].

FDG-PET in DLB shows generalised low uptake and reduced occipital hypometabolism [118, 119]. Larger studies however have shown that FDG-PET has sensitivity of 74% and specificity of 70% for DLB and therefore cannot be used as an indicative biomarker [21, 110]. Another characteristic pattern of FDG-PET in DLB is the relative preservation of the posterior or mid-cingulate metabolism, the so called ‘cingulate island’ sign which has been shown to have particular prognostic value, especially when evaluated using semi-quantitative computerised image analyses [120–122]. Scans from DLB patients with presence of co-existing AD pathology are much less likely to show the cingulate island sign [123]. Using spatial covariance analysis of FDG-PET data, Ingram et al. were able to discriminate AD from DLB with an AUC of 0.84 [124].

FDG-PET in VaD showed reduced uptake in deep grey structures, the cerebellum, the middle temporal gyrus and the anterior cingulate compared to AD [125]. In FTD, FDG-PET has been associated with frontal hypometabolism [126, 127] in the early stages with the progression of the disease also affecting the parietal and temporal cortices [128]. FDG-PET has significant diagnostic accuracy for the differential diagnosis between AD and FTD with high specificity (>95%) in multiple studies [129–131]. FDG-PET performed in 52 patients with suspected bvFTD but not having characteristic atrophy patterns on structural imaging was 47% sensitive and 92% specific, showing that is able to identify nearly half of the patients with bvFTD undetected by MRI with high specificity, enabling exclusion of psychiatric and other neurodegenerative disorders [132]. Among 548 subjects with different types of dementia including 110 healthy elderly, 114 MCI, 199 AD, 98 FTD and 27 DLB, FDG-PET was able to correctly classify 95% of AD, 92% DLB, 94% FTD and 94% of the healthy elderly [129]. Similar to young-onset AD, in genetic forms of FTD, hypometabolism on FDG-PET can be seen at least 10 years before symptom onset [133] and the higher severity of symptoms in these forms correlates with more widespread areas of hypometabolism on FDG-PET [134]. Tripathi et al. performed FDG-PET in 101 patients with a clinical diagnosis of dementia and showed that FDG-PET was concordant with the clinical diagnosis of dementia (confirmed with 8-month follow up) in 90% of patients scanned (93.4% for AD, 88.8% for FTD, 66.6% for DLB and 92.3% for other dementia syndrome) [135]. A Delphi consensus expert panel reviewing the available literature on FDG-PET for the differential diagnosis of AD, DLB, FTD, VaD and non-degenerative pseudodementia concluded that although there is lack of evidence on which to base strong recommendations, FDG-PET is useful in the differential diagnosis of dementia but require additional prospective studies in patients with diagnostic uncertainty [136].

Conditions such as PSP, autoimmune encephalitis, chronic schizophrenia, alcohol related brain damage and late onset psychiatric disorders [137–139] may also be associated with patterns of frontal hypometabolism on FDG-PET and therefore FDG-PET should be used in combination with history and other examinations available [140–142].

Importantly one of the limitations of FDG-PET seems to be the fact that it is inversely affected by brain glycaemia, suggesting that in diabetic patients caution would be required to interpret significant findings [143]. In summary, FDG-PET is a useful tool in clinical practice as is sensitive and specific for the diagnosis of established AD and other dementias but its role in the early and MCI stages is limited due to lack of sensitivity.

## Imaging markers for synucleopathies

Brain dopamine transporter imaging using ^123^I-Ioflupane (FP-CIT) SPECT and cardiac sympathetic nerve imaging using ^131^I-Metaiodobenzylguanidine myocardial scintigraphy (MIBG) are well established markers for the diagnosis of DLB with high sensitivity and specificity and both are indicative biomarkers as part of the current International Consensus diagnostic criteria for DLB [21, 144–146]. A large multi-site study showed that FP-CIT SPECT differentiated DLB from AD with 78% sensitivity and 91% specificity while a further autopsy study showed that FP-CIT has accuracy of 86% (sensitivity 80%, specificity 92%) compared to neuropathological diagnosis of DLB [145, 147]. Dopaminergic imaging can be abnormal in other neurodegenerative disorders where dopaminergic transmission is affected, such as FTD, CBD, PSP and MSA [148–150]. Regarding early diagnosis, a study in 144 patients with MCI showed that that FP-CIT scans are 76% accurate (sensitivity 66%, specificity 88%) for probable MCI-DLB suggesting that dopaminergic imaging is useful at the MCI stage where LBD is suspected [151]. Several other radioligands for aspects of the dopaminergic system have been tested involving both pre- and post-synaptic processes with the potential for use in clinical practice and therapeutic trials but require more research in larger and cross-diagnostic cohorts [152].

## Amyloid PET imaging

Amyloid PET has been established as an important imaging tool in the early, specific and unbiased diagnosis of AD [153–155]. It is part of the biomarker screening in the AD diagnostic criteria and can help particularly in the diagnosis of young onset AD and differentiate from other dementias such as bvFTD [156, 157]. There are several amyloid PET tracers currently available for use, namely ^11^C-Pittsburgh compound–B (PiB), ^18^F-flutemetamol, ^18^F-florbetaben, ^18^F -florbetapir that image Aβ plaques and have been validated through autopsy studies [158–162]. Amyloid PET is either assessed using visual rating assessment or through quantification methods using the standard update value ratio (SUVR) or the centiloid scale, the latter providing a common framework for assessing amyloid uptake whichever PET ligand is used [163]. Serial amyloid PET scans show that Αβ deposition starts in the anterior temporal areas and spreads to the frontal and medial parietal areas, the associative neocortex and later on at the primary sensorimotor areas and subcortical regions mirroring the neuropathological staging of AD pathology [164–166]. Studies have suggested that amyloid PET provides incremental diagnostic value beyond clinical and FDG-PET diagnoses of AD. Compared to FDG-PET, amyloid PET is more sensitive (89% vs 73%) but less specific (83% compared to 98%) for the differential diagnosis between AD and FTD [167, 168].

Amyloid PET has helped to understand the temporal relationship between Aβ and tau in vivo and has shown that Aβ deposition begins several decades before symptom onset. In epidemiological cohorts assessing patients without dementia using amyloid PET status or CSF profile, Aβ pathology was associated with APOE genotype, presence of cognitive impairment and suggested a 2- to 30- year interval between first development of Αβ positivity and onset of dementia [3, 169]. Amyloid PET positive status in 69 cognitively normal, 52 MCI and 31 AD was shown to be associated with greater cognitive and global deterioration over a 3 year follow up compared to amyloid PET negative subjects showing prognostic potential [170]. Serial amyloid PET is promising in the prediction of cognitive decline in initially cognitively unimpaired individuals with a cluster of precuneus, lateral orbitofrontal and insular regions showing the particular associations [171]. Anti-Aβ immunotherapies have shown reductions in amyloid PET and several amyloid reducing therapies for AD use amyloid PET (or CSF) as a key endpoint [172, 173].

Αβ pathology is present in ~50% of DLB patients and the use of amyloid PET cannot be used for the differential diagnosis of AD and DLB, that are two of the most common types of dementia [174]. In DLB there are no clear differences in clinical symptoms and disease severity between amyloid PET positive and negative status [175]. Amyloid PET has however been associated with cortical thinning in the hippocampus and greater grey matter loss in the cingulate gyrus and temporal lobe in DLB and this may be suggestive of faster neurodegeneration and worse clinical progression in positive Aβ LBD compared to negative status [46, 174, 176].

The use of amyloid PET imaging has several limitations as it does not correlate with symptom onset and disease severity, cannot predict time of onset of dementia syndrome while its use in older populations seem to require more research as amyloid pathology is prevalent in a large proportion of elderly cognitively unimpaired individuals [177]. In summary, amyloid PET is an important tool for the specific diagnosis of AD in the early and preclinical stages, has revolutionised our understanding of the chronology of AD pathophysiology and has opened new therapeutic windows for identification of people more at risk of getting AD and for use in therapeutic AD trials.

## Tau PET imaging

Tau PET uses ligands that bind to neurofibrillary tangles. Tau PET is approved by the FDA for the in vivo assessment of tau in people with AD [178]. There are several tau PET tracers that have been developed so far with the initial first generation (^18^F-AV1451/now licensed for clinical use in US as flortaucipir, ^18^F-THK family and ^11^C-PBB3) and the newer tracers ^18^F-MK6240, ^18^F-R0948, ^18^F-PI260, ^18^F-GTP1, and ^18^F-JNJ-64326067 [178, 179]. The initial tau PET tracers showed good specificity for cortical tau tangles however there has been evidence of non-specific binding in subcortical structures suggesting that tau PET with these tracers is not suitable for non-AD tauopathies [179–183]. Studies combining antemortem imaging with neuropathological validation have shown that ^18^F-flortaucipir PET is highly sensitive for presence of high levels of AD neuropathological change with visual rating positivity corresponding to Braak levels IV or greater [184, 185]. Second generation tau PET tracers have shown greater promise in detecting earlier Braak stages while show different properties in non-AD tauopathies [186, 187].

**Fig. 3:**
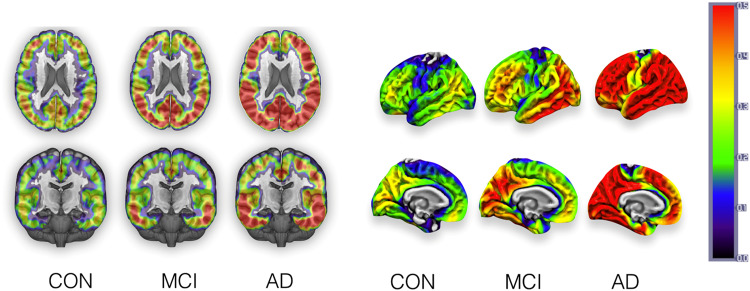
Tau Positron emission tomography in Mild Cognitive Impairment (MCI) and Alzheimer’s Disease (AD). The figure shows the stereotypical progression of tau binding on 18F-flortaucipir from normal controls (CON) to MCI to AD depicting volume and surface in the left and right panels respectively. These are group-averaged mean maps from the NIMROD study cohort. The top of the coloured bar (red) signifies greater radioligand binding and the bottom of the bar (blue/grey) lower binding. Images are courtesy of Dr Elijah Mak, University of Cambridge, UK.

Tau PET correlates with patterns of tau pathology deposition and shows strong correlations with cognitive function, even in cognitively normal older people [188]. Longitudinal deposition in tau PET is associated with baseline levels tau and Aβ deposition is a necessary antecedent for spread of tau beyond the temporal lobe in AD [189, 190] (Fig. 3).

In a cohort of 20 AD and 15 controls who underwent ^18^F-flortaucipir, ^11^C-PiB and FDG-PET, regional tau PET deposition co-localised with hypometabolic regions and correlated with areas critical for cognitive functions uniquely affected in distinct variants of AD (e.g. posterior cortical atrophy) [191]. In a multicentre cross-sectional study that included 719 participants (150 controls, 126 MCI, 170 AD and 254 other neurodegenerative disorder), ^18^F-flortaucipir PET had 89.9% sensitivity and 90.6% specificity in discriminating AD from non-AD neurodegenerative disorders and higher AUC (0.92–0.95) compared to volumetric MRI measures (AUC 0.63–0.75) [192]. It did show however slightly lower accuracy (AUC 0.75–0.84) when comparing positive amyloid PET MCI to other non-AD neurodegenerative disorders [192]. In a longitudinal multicentre prognostic study with 1431 participants (673 cognitively unimpaired, 443 MCI and 315 AD all with positive Aβ status), baseline tau PET (^18^F-flortaucipir or ^18^F-FRO948) could predict change in mini-mental state examination (MMSE) over a period of almost two years and outperformed amyloid PET and MRI volumetric measures, suggestive that tau PET has a prognostic value in preclinical and prodromal AD [193]. Tau PET, in a cohort of 1612 individuals, has helped to understand the trajectories of tau pathology spread, showing four distinct patterns including a limbic-predominant, a medial-temporal lobe sparing pattern and posterior and lateral temporal patterns associated with atypical clinical variants of AD [194].

Apart from accurate diagnosis of AD, tau PET is likely to play an important role in future therapeutic trials both in terms of subject stratification on entry and measuring longitudinal deposition of tau and any changes in the rate of accumulation or reduction in pathology may be used as outcomes in therapeutic trials [195]. A study aggregating results from two Phase II Clinical trials that have used ^18^F -flortaucipir PET scans in a total of 364 study participants showed that tau PET provides valuable prognostic information in terms of clinical deterioration over 18 months in MCI and AD [196]. Another study in 32 early AD participants showed that tau PET but not amyloid PET predicted the rate of subsequent brain atrophy showing that tau pathology is likely a major driver of neurodegeneration and has a role in precision medicine and future trials [197].

Α study using ^18^F-flortaucipir in 10 DLB compared to 27 AD and 14 controls showed minimal deposition of ^18^F-flortaucipir in DLB, with medial temporal lobe ^18^F-flortaucipir being able to distinguish DLB from AD with an AUC of 0.87 [198]. Overall, tau PET deposition mirrors the progression of AD pathology, correlates with disease severity and in that respect it is hypothesised that will become one of the most important tools for the differential diagnosis between AD and non-AD dementias [191, 199, 200].

## Other important PET ligands

**Fig. 4:**
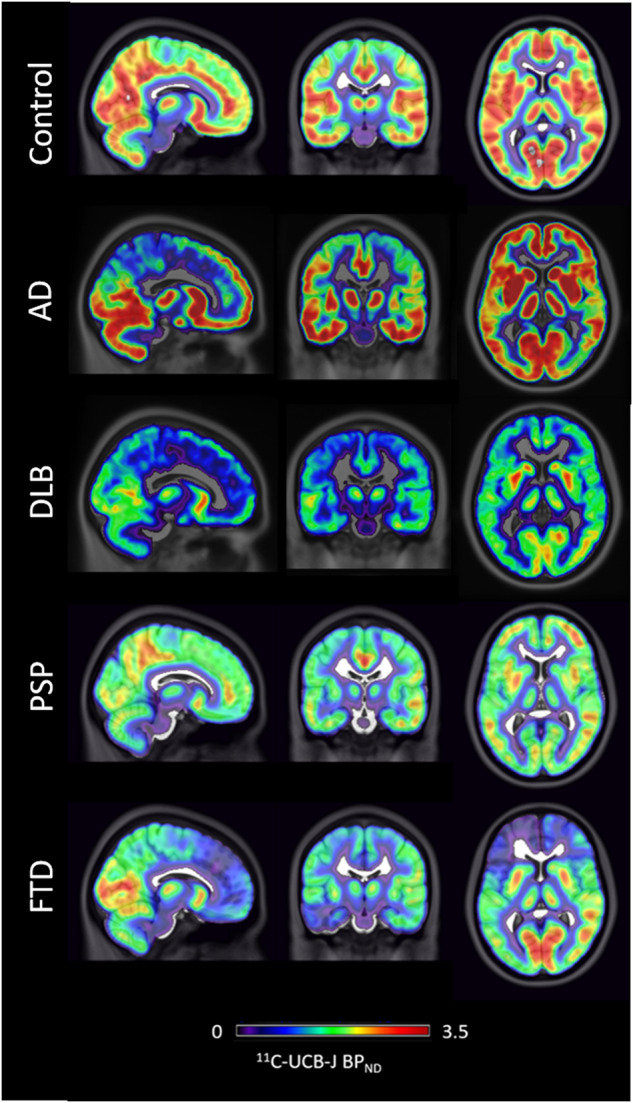
Synaptic PET imaging in different types of dementia. ^11^C-UCBJ PET cases images showing differential loss of synaptic density in cases of people with Alzheimer’s disease (AD), Dementia with Lewy Bodies (DLB), Progressive supranuclear palsy (PSP) and Frontotemporal dementia (FTD) compared to a healthy control. Images are courtesy of Dr Maura Malpetti and Dr Simon Jones, University of Cambridge, UK.

Aside from the latest developments in amyloid PET and tau PET, several other PET ligands have been investigated in dementia, both to explore their involvement in underlying pathophysiology and as early diagnostic markers. A number of studies have used the binding potential of ^11^C-UCBJ PET which binds to the synaptic vesicle protein 2a (SV2A) as a marker of synaptic density [201]. In parallel a fluoride ligand measuring synaptic density ^18^F-SynVesT-1 has been recently developed [202]. One of the first studies in ^11^C -UCBJ PET comparing 11 controls with 10 AD showed reduction in hippocampal binding in keeping with neuropathological findings in AD [203]. A follow up study in 34 early AD and 19 controls confirmed reduction of ^11^C-UCBJ PET binding in medial temporal and neocortical brain regions in early AD [204] while severe synaptic loss has been observed using ^11^C -UCBJ PET in series of cases with DLB, FTD, PD, PDD, PSP, CBD and C9Orf72 mutation carriers [205–209]. Nevertheless, such groups have not yet been compared directly within the same study to characterise differences in regional distribution and provide discrimination accuracy statistics or disease specific maps. ^11^C-UCBJ PET has shown inverse correlation with tau PET deposition in amnestic MCI as well as in PSP and CBD [210, 211]. In a study with 14 AD and 11 cognitively normal participants, ^11^C -UCBJ PET reductions reflected FDG-PET changes in the medial temporal regions, while in neocortical regions FDG-PET showed greater reductions compared to ^11^C-UCBJ PET [212]. Further studies will need to address such important preliminary findings in the large scale, test the timing of onset of in vivo synaptic changes and whether they can be used for early and differential diagnosis. While large multi-centre studies across multiple diagnostic groups as well as longitudinal studies are awaited, an in vivo marker for synaptic density is a promising readout for early results of therapeutic trials that may target preservation and restoration of brain synapses (Fig. 4).

Another important PET-imaging modality in dementia is the in vivo imaging of neuroinflammatory processes [213, 214]. One of the earliest and the most widely used PET markers of neuroinflammation in neurodegenerative studies is ^11^C -PK11195 (PK-PET) which binds to the 18-kDA translocator protein (TSPO), a mitochondrial membrane protein that is upregulated in activated microglia [215]. PK-PET is increased in the entorhinal, temporal, parietal and cingulate cortex in AD, in the frontotemporal regions in FTD, while PSP shows increased PK-PET binding in the thalamus, putamen and pallidum, showing differential distribution of neuroinflammatory processes in different types of dementia [215–218]. In DLB, microglial activation using PK-PET was shown to be occurring early in the disease in key regions associated with the pathology [219]. Longitudinal studies using PK-PET in AD have shown a longitudinal increase in microglia activation with a positive correlation with amyloid PET deposition and inverse correlation with FDG-PET [220], while baseline PK-PET in AD and PSP was able to predict clinical progression [221, 222] and was associated with small vessel disease, particularly hypertensive arteriopathy in AD [223]. PET markers of TSPO are influenced by TSPO genotype and cannot distinguish the contribution of different glial cells, for example is unclear to what extent astrocytes influence signal [224]. Considering the limitations of PK-PET, several other markers of TSPO are being developed and tested in dementia such as ^11^C-PBR28, ^11^C-DAA1106 (for review see [225]). Using ^11^C-PBR28 Ferrari-Souza et al. found that APOE ε4 carriers present with increased microglial activation early in AD and independent of amyloid [226]. While work in understanding the role of neuroinflammation using in vivo PET imaging is ongoing, larger multi-centre studies directly comparing diagnostic groups as well as longitudinal studies are lacking, while newer PET markers of neuroinflammation, such as PET imaging of the P2x7 receptor and the colony-stimulating factor 1 receptor, are being developed to try and improve signal to noise ratio and higher binding affinity [213, 227–229]. Studies have also focused on PET imaging of reactive astrocytes as proxy for neuroinflammation ([230, 231]). Reactive astrogliosis, the activation and transformation of astrocytes during disease, has been implicated in the early stages of AD and in vivo PET biomarkers of reactive astrogliosis have been tested across the AD continuum [232–234]. The PET ligand ^11^C-DED which captures changes in astrocytic monoaminoxidase-B density has been found increased in AD [230, 235]. A newer tracer ^11^C-BU99008 which binds to I-imidazoline in the astrocyte mitochondrial membrane appears to have a region specific association with PET amyloid with positive correlations in the primary motor and sensory areas and negative association in temporal lobe and cingulate cortices [236, 237] Another study using ^11^C-BU99008 along with FDG-PET, amyloid PET and MRI showed that patients with a positive amyloid scan showed greater astrocyte reactivity and showed that particularly regions representing earlier stages of pathological progression with lower amyloid have increased astrocyte reactivity while regions with more advanced disease progression show reduced astrocyte reactivity [238]. While such studies show huge potential they still require further development of more specific tracers to be tested in larger cohorts [231].

Several other studies have explored the role of a variety of PET ligands in dementia, for example imaging the cholinergic system showing cholinergic transmission loss in AD and even higher changes in LBD [239–242]. A number of other novel PET tracers are in development [243]. For example a recent study tested the potential of ^11^C -Martinostat PET that binds to the epigenetic molecules of class I histone deacetylaces, showing that they are reduced in patients with AD and mediate the effects of Aβ and tau on brain atrophy and cognitive impairment in keeping with post-mortem studies [244]. A large volume of research is focusing on imaging of the locus coereleus region as the brain’s source of norepinephrine and a new ^18^F^-^Fluorotyrosine PET ligand has been developed to measure catecholamine synthesis [245]. This was studied in cognitively normal adults showing associations with tau PET but has not yet been tested in dementia patients [245].

## Conclusions

In summary, decades of brain imaging research in dementia has revolutionised our understanding of the different conditions causing dementia and changed routine clinical practice with neuroimaging now firmly established in clinical diagnostic pathways. Brain imaging is key for the early, accurate and differential diagnosis of dementia. An expert consensus has recommended three pathways in situations where further testing is warranted for the clinical diagnosis of dementia. The first pathway involves use of Amyloid PET or CSF if AD is suspected. Second, FDG-PET could be used when the initial workup suggests a non-AD dementia and third in the cases of cognitive problems with movement disorder then a FP-CIT SPECT or MIBG could be used [154]. Importantly, brain imaging is increasingly used as means for stratification of participants for clinical trials and as a marker of treatment response in disease modifying therapeutic trials.

New developments in advanced multimodal MRI imaging along with wide use of PET imaging with specific ligands linked to pathology are now being tested in large longitudinal multi-centre cross diagnostic cohorts. This will allow their translation to clinical practice. With the evolution of neuroimaging techniques and their adoption in clinical practice, additional challenges will arise with the computational requirements for novel methods such as DTI or calculation of longitudinal brain changes. Nevertheless, considering the complexity and variability of the different types of dementia along with the presence of mixed pathologies, machine learning algorithms, such as the Subtype and Stage inference (Sustain) have been introduced to discover data-driven disease phenotypes with distinct temporal progression patterns [246]. The Sustain algorithm can identify phenotypes in genetic FTD from imaging alone while in AD it has uncovered three subtypes opening opportunities for disease subtype phenotyping and better precision medicine [246]. The use of such advanced analytic methods in the future may help to better integrate multiple layers of data such as genetics, fluid biomarkers and neuroimaging in order to improve the current framework for understanding and staging the different types of dementia.

Brain imaging has helped to improve the biological classification of AD by allowing proposed classifications based on pathology, like the ATN staging, to be applied in vivo and opened a new window of therapeutic intervention at preclinical stages. Such imaging has also improved our understanding of the role of mixed pathologies in dementia [102, 175, 189, 247].

With the addition of advance analytic strategies to support the analytic methods, there is potential of more accurate diagnostics while the development of specific markers, for example obvious key gaps for dementia are ligands for α-synuclein and TDP-43 pathology [248–251].
